# Fiscal Decentralization and Local Environmental Pollution in China

**DOI:** 10.3390/ijerph17228661

**Published:** 2020-11-21

**Authors:** Shufen Guo, Ludi Wen, Yanrui Wu, Xiaohang Yue, Guilian Fan

**Affiliations:** 1 Cooperative Innovation Center for Transition of Resource-Based Economies, Shanxi University of Finance and Economics, Taiyuan 030006, China; sdguosf@163.com (S.G.); fanguilian@163.com (G.F.); 2 School of Business Administration, Shanxi University of Finance and Economics, Taiyuan 030006, China; 3 Department of Economics, University of Western Australia, Nedlands 6907, Australia; yanrui.wu@uwa.edu.au; 4 Sheldon B. Lubar School of Business, University of Wisconsin-Milwaukee, Milwaukee, WI 53201-0413, USA; xyue@uwm.edu

**Keywords:** fiscal decentralization, government environmental preference, environmental pollution, moderating effect, threshold effect

## Abstract

Fiscal decentralization is one of the tools for the central government to engage local governments in environment management. However, its effects are inconclusive. This paper aims to examine the impact of fiscal decentralization on environmental pollution and the role of government environmental preference in China’s provinces. The results show that fiscal revenue decentralization exacerbates local environmental pollution more seriously than expenditure decentralization. This negative environmental effect of fiscal decentralization could be moderated by government environmental preference. Based on our findings, it is recommended that China’s local governments should improve environmental preference so that fiscal decentralization can create a win–win situation for the economy and environment. Furthermore, the different effects of fiscal revenue and expenditure decentralization create a necessity for differentiated management of fiscal decentralization by the central and local governments.

## 1. Introduction

In recent years, governments of all levels in China have emphasized environmental governance and ecological reconstruction. In 2014, six clauses in China’s Environmental Protection Law promulgated by the central government mentioned “finance”, indicating the determination of the central government to use fiscal methods to control environmental pollution. Fiscal decentralization defines the relationship between central and local government finances and reflects local government financial autonomy in both revenue and expenditure. From a fiscal perspective, investment in energy conservation and environmental protection is the main initiative for local governments to participate in environmental protection. To a certain extent, fiscal expenditure in environmental protection reflects the environmental preference of a local government [1]. However, due to competition among local governments and the mechanism of promoting officials on the basis of GDP performance in China, local governments generally prefer economic growth to environmental protection [2,3], and hence the actual implementation of local environmental policies may be compromised.

Under the current fiscal decentralization system in China, the central government obtains fiscal revenue from local governments for macro-control, while it also allows a certain degree of fiscal decentralization for local governments. As the largest developing country in the world, the practice in China may provide implications for other developing countries. In fact, the Chinese government has already taken some action towards it. In 2018, in order to motivate local governments to take the main responsibility for pollution control, the pollution discharge fee was changed to the environmental protection tax which is all maintained as local fiscal revenue. In addition, in order to encourage enterprises to reduce pollutant emissions, more tax deduction is linked with the amount of emissions reductions. In terms of fiscal expenditure, since 2007, the proportion of central transfer payments in local environmental protection expenditures has played a leading and exemplary role [4].

The purpose of this paper is to answer the following questions. Will China’s fiscal decentralization exacerbate local environmental pollution? Will the environmental effects of fiscal decentralization be affected by government environmental preference? To address these problems, we first analyze the environmental effects of two types of fiscal decentralization, namely revenue decentralization and expenditure decentralization. Second, we check the robustness of our results by considering alternative indicators of pollution. The main contribution of this paper is to incorporate government environmental preference into the analysis of fiscal decentralization and local environmental pollution. Such analysis not only provides empirical evidence from China for research in environmental federalism [5] of developing countries, but also develops a sustainability-oriented [6] fiscal and environmental governance. The remaining content of this paper is organized as follows. Section 2 introduces the theories of fiscal decentralization and environmental pollution as well as the research hypotheses. Section 3 describes the data and models used in the analysis. The empirical evidence and scientific discussion are described in Section 4. In this section, a fixed effect model and a threshold effect model are used to explore the effect of fiscal decentralization and government environmental preference on environmental protection. Section 5 concludes the paper.

## 2. Theoretical Analysis and Research Hypotheses

Studies of fiscal decentralization have focused on its economic [7,8], ecological [9], and social [10] effects. However, there is no consensus about the effects of fiscal decentralization on environmental pollution. The impact of fiscal decentralization on environmental governance is generally divided into two categories, namely “race to the top” and “race to the bottom”. Environmental federalism believes that environmental protection is closely related to the decentralization of the government [5]. The first generation of fiscal decentralization theory believes local governments have a clear information advantage over the central government in terms of local realities [11]. Therefore, environmental protection requires local governments to take actions based on local conditions [12]. However, other studies show that externality of fiscal policy may result in inefficient local public spending [13]. The second generation of fiscal decentralization theory assumes that the local governments be essentially “rational people”, which means that the local officials are likely to sacrifice public welfare to pursue career promotions or fiscal revenue [14].

Because of different models and indicators for environmental pollution, empirical studies are mixed [15]. List and Gerking (2000) provided evidence that fiscal decentralization does not cause a decline in environmental quality [16]. After Reagan’s decentralization policy, the environmental quality is consistent with decentralization leading to a race to the top in the United States [17]. While the cross-country estimates showed that fiscal decentralization increases pollution for more than 80 developed and developing countries [18], Fredriksson et al. (2006) confirmed the negative effect of fiscal decentralization on environmental policy using cross-sectional developing country data, in particular for air pollution policies [19]. Studies on environmental effects of China’s fiscal decentralization have also achieved some mixed results. Using the principle of utility maximization and spatial econometric models, Kuai et al. (2019) confirmed that both fiscal revenue and expenditure decentralization have positive effects on the decline of concentration of PM2.5, i.e., particle matter smaller than 2.5 microns [20]. He (2015) considered system GMM (Generalized method of moments) estimation and showed that fiscal decentralization has no significant effect on per capita emissions of wastewater at all but a significantly positive effect on pollution abatement spending and pollutant discharge fees [21]. Other authors argue that fiscal decentralization exacerbates environmental pollution. In terms of carbon emissions, an increase in fiscal decentralization is not conducive to the reduction in environment pollution [22]. Due to local government competition, officials tend to prefer infrastructure construction to environment protection and other public service construction which aggravates environmental pollution [23].

The fiscal budget expenditure of a province is roughly equal to the sum of its budget revenue, extrabudgetary revenue, and net transfer payments from the central government [9]. Since the reform of the tax system in China in 1994, fiscal expenditure decentralization has been far greater than fiscal revenue decentralization [4] Therefore, local governments need to assume more responsibilities in the provision of public goods and services. From the revenue perspective, the higher the degree of fiscal decentralization, the greater power of controlling fiscal revenue local governments have. In order to achieve economic growth and increase local fiscal revenues, local governments may relax environmental regulations to support the enterprises with high pollution and high energy consumption [24]. However, from the expenditure perspective, the higher fiscal decentralization, the more responsibilities that local governments ought to assume. Because the fiscal expenditure of a province is always larger than the fiscal revenue, the difference is generally met by the central government’s fiscal transfer payment. Under the current fiscal decentralization system, local governments compete for economic performance. This system leads to a fiscal expenditure structure distortion. Infrastructure items are emphasized while non-productive expenditure items are ignored [25]. Fiscal decentralization will encourage local governments to relax environmental regulations and reduce their efforts to protect the environment, and thus accelerate the local environmental pollution [22]. Since fiscal revenue decentralization and fiscal expenditure decentralization have different effects on local government behavior, it is necessary to explore their impact on local environmental pollution separately. The above analysis of the existing literature leads to our first hypothesis.

**Hypothesis** **1.** *Fiscal decentralization has a negative impact on reducing environmental pollution.*

While studying the relationship between fiscal decentralization and regional innovation efficiency, government innovation preference (the proportion of scientific and technological expenditure in local fiscal expenditure) has a mediating effect in some regions in China [26]. Similarly, government environmental preference (the proportion of environmental protection expenditure in local fiscal expenditure) has a mediating effect on the relationship between fiscal decentralization and environmental governance efficiency [1]. This paper draws on these ideas and introduces government environmental preference into the relationship between fiscal decentralization and environmental pollution. Government environmental preference is measured not only by the proportion of environmental protection expenditure in local fiscal expenditure but also by per capita fiscal environmental protection expenditure. 

There is an unclear complex mechanism in the impact of fiscal decentralization on the environment. On the one hand, although there are interactions of fiscal decentralization with other factors, like economic growth [23], the influence of government environmental protection expenditure on the environmental effect of fiscal decentralization is unclear. Empirical results about this issue are mixed. Wu and Wang (2018) showed that, in 73 key monitoring cities in China, although environmental protection expenditure can ease the negative impact of fiscal decentralization on reducing haze pollution, its effect is trivial [27]. Zhang (2018) also illustrated that, among three environmental protection policies of local governments, environmental protection expenditure does not affect the environmental effect of fiscal decentralization [8]. On the other hand, Hong et al. (2018) demonstrated that there is a significant threshold effect of environmental fiscal policy on local environmental pollution [28]. In fact, since Grossman and Krueger (1991) first proposed that the relationship between SO_2_ emissions and per capita GDP is an inverted “U” shape [29], more and more researchers agree that the influence of economic factors (including fiscal factors) on environmental pollution is non-linear. This paper assumes that the environmental effect of fiscal decentralization is affected by government environmental preference. In other words, as government environmental preference increases to a certain level, the negative impact of fiscal decentralization on the environment will ease. According to the above analysis, we put forward our second hypothesis.

**Hypothesis** **2.** *Government environmental preference can offset the negative environmental effect of fiscal decentralization.*

## 3. Research Design

### 3.1. Description of the Models and Variables

To verify the two hypotheses proposed, this study constructs three models. First, the following panel fixed effects model is proposed to examine the direct impact of fiscal decentralization on local environmental pollution.

(1) $${EP}_{it}{\;=\;\alpha }_{0}{\;+\;\alpha }_{1}{FD}_{it}\;+\;\sum \alpha_{j}X_{ijt}{\;+\;\mu }_{i}{\;+\;\nu }_{t}{+\varepsilon }_{it}$$

where *EP*, *FD* and *X* represent environmental pollution, fiscal decentralization and control variables. $\alpha_{1}$ is the core coefficient which indicates the impact of fiscal decentralization on local environmental pollution. *μ* is the regional effect, *ν* is the time effect and *ε* is error term. *i* and *t* denote the provinces and years, respectively. Through this framework we somewhat control the unobservable heterogeneity.

Environmental pollution (*EP*) is expressed as the ratio of industrial SO_2_ emission to the land area or industrial SO_2_ emission density. China’s energy endowment is dominated by coal. Therefore, SO_2_ is one of China’s major pollutants and the key target of industrial pollution control [30]. Furthermore, industrial SO_2_ is one of the key pollutants monitored and recorded by China’s environmental protection authorities. The environmental control policy issued by the Chinese government in 1997 targets the geographic density of SO_2_ emission. For these reasons, the density of industrial SO_2_ emissions is chosen as the indicator of the local environmental pollution [31]. To take land scale and economic size into account, seven provinces in minority areas (Qinghai, Guizhou, Yunnan, Guangxi, Inner Mongolia, Xinjiang and Ningxia) are removed from the sample to form an alternative sample [32]. On the one hand, these provinces are vast regions with relatively small economies due to the geological conditions, so using land area as a denominator to calculate SO_2_ emission density may be biased [23]. On the other hand, the fiscal expenditure decentralization in minority areas is generally overestimated [33], because the central government’s transfer payments to minority areas are generally much greater than those to other areas [34]. Later, for the robustness check, we use another indicator, NO_X_ emission density, as an alternative explanatory variable.

The key independent variable is the degree of fiscal decentralization (*FD*). The higher degree of fiscal decentralization, the more autonomy the local governments have to allocate fiscal resources. In this paper, we consider both fiscal revenue decentralization (*FD1*) and fiscal expenditure decentralization (*FD2*). China’s local government revenue consists of two components, revenue generated locally and central government’s transfer payment and tax refunds. We follow the existing literature and use the ratio of local fiscal revenue (expenditure) to central fiscal revenue (expenditure) as an indicator of fiscal decentralization [7,21]. Subsequently, to control the possible correlation between population size and fiscal revenue or expenditure, fiscal decentralization is measured by the ratio of per capita fiscal revenue (expenditure) in a province to per capita fiscal revenue (expenditure) of central government.

There are four control variables in this model. The economic development (*GDP*) indicator is expressed by GDP per capita. A large number of studies have shown that the level of economic development is an important factor influencing the environmental quality [29,35,36]. The degree of openness (*FDI*) is expressed by the ratio of the foreign direct investment to GDP. On the one hand, looser environmental regulation and standards in the host country can attract foreign direct investment. However, the large amount of foreign capital inflow will bring pollution industries and worsen the quality of environment in the host country. On the other hand, foreign-funded enterprises may have more advanced production and pollution treatment technology, which is conducive to pollution reduction and environmental quality improvement [33]. Science and technology (*ST*) are expressed by the number of patents granted per 10,000 persons. Regions with higher R&D investment may have more advanced production technologies to control environmental pollution [37]. The industrial structure (*IS*) is expressed as the ratio of the added value of the secondary industry to the total output value of the region. Differences in regional industrial structure often lead to different levels of local environmental pollution. Compared with the tertiary industry, the secondary industry is more likely to cause environmental pollution [38].

Second, to test Hypothesis 2, model (1) is extended to incorporate the government environmental preference as the moderator variable. We introduce the interaction term between the government environmental preference and fiscal decentralization to test the moderating effect of environmental preference in the regression. In order to reduce the endogeneity issues, the government environmental preference is lagged by one year in fixed effect models.

(2) $${EP}_{it}{\;=\;\beta }_{0}{\;+\;\beta }_{1}{FD}_{it}{\;+\;\beta }_{2}{GEP}_{i,\;\mathrm{t-}1}{\times FD}_{it}\;+\;\sum \beta_{j}X_{ijt}{\;+\;\mu }_{i}{\;+\;\nu }_{t}{\;+\;\varepsilon }_{it}$$

where GEP represents government environmental preference and other variables are the same as in model (1). In model (2), if $\beta_{1}$ and $\beta_{2}$ are significant and have the opposite sign, it indicates that government environmental preference can offset the environmental effect of fiscal decentralization.

Government environmental preference is measured by per capita fiscal environmental protection expenditure (GEP1) as well as the proportion of environmental protection expenditure in local fiscal expenditure (GEP2). In 2007, China’s Ministry of Finance began reporting fiscal environmental protection expenditure through the China Financial Yearbook. China’s environmental protection investment is still dominated by government spending. The environmental protection expenditure as part of local government fiscal expenditure is an important quantitative indicator of local governments’ attitude towards environmental protection [8]. More environmental protection expenditure implies greater environmental preference of the local government.

Third, we further explore the threshold effect of government environmental preference. The model for two threshold values is shown as follows.

(3) $$\begin{array}{ll} {EP}_{it}{\;=\;\gamma }_{0}\;+ & \gamma_{1}{FD}_{it}\;\times \;I\left({GEP}_{it}\;\leq {\;\tau }_{1}\right){\;+\;\gamma }_{2}{FD}_{it}\;\times \;I\left(\tau_{1}{\;<\;GEP}_{it}\;\leq {\;\tau }_{2}\right)\\ & {+\;\gamma }_{3}{\mathrm{FD}}_{\mathrm{it}}\;\times \;I\left(\tau_{2}{\;<\;GEP}_{it}\;\right)+\sum {\;\gamma }_{j}X_{ijt}{+\;\mu }_{i}{+\nu }_{\;t}{+\;\varepsilon }_{it} \end{array}$$

where {*τ*} are the threshold values, and other variables are the same as in models (1) and (2). In model (3), if regression coefficients ($\gamma_{1}$,${\;\gamma }_{2}$,$\;\gamma_{3}$) of fiscal decentralization are significant, it indicates that the influence of fiscal decentralization on local environmental pollution changes as government environmental preference varies.

### 3.2. Data

The China Financial Yearbook has listed government environmental protection expenditures since 2007, and the China Environmental Statistics Yearbook released industrial SO_2_ emissions up to 2015. Due to these constraints, this study considers a panel data of 30 Chinese provinces from 2007 to 2015. The descriptive statistics of the variables are shown in Table 1. All the economic indicators are converted into 2006 constant prices.

### 3.3. Preliminary Analysis

**Figure 1:**
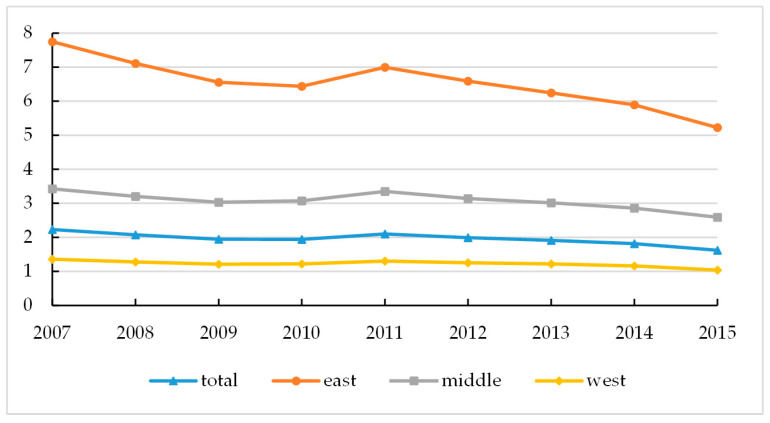
China’s industrial SO_2_ emissions density in 2007–2015 (*y*-axis unit: tons/km^2^).

During the period 2007–2015, China’s industrial SO_2_ emissions density peaked in 2011. The increase in industrial SO_2_ emission density in 2009–2011 may be the consequence of economic stimulus in response to the global financial crisis in 2008. Figure 1 shows that industrial SO_2_ emission density in the eastern region is much higher than that in the central and western regions. However, the decline in the industrial SO_2_ emission density in the eastern region is also sharper than the central and western regions. It dropped from 7.75 tons/km^2^ in 2007 to 5.23 tons/km^2^ in 2015, which to some extent reflects the upgrading of the industrial structure in the eastern region. In addition, the industrial SO_2_ emissions density has declined in all regions since 2011. Interestingly, China’s total energy consumption (10,000 tons of standard coal) increased by one-third from 2007 to 2015, while the industrial SO_2_ emission density dropped from 2.23 tons/km^2^ to 1.62 tons/km^2^. This indicates that China has upgraded desulfurization due to both technological progress and policies for promoting emission reduction targets and emission standards. 

**Figure 2:**
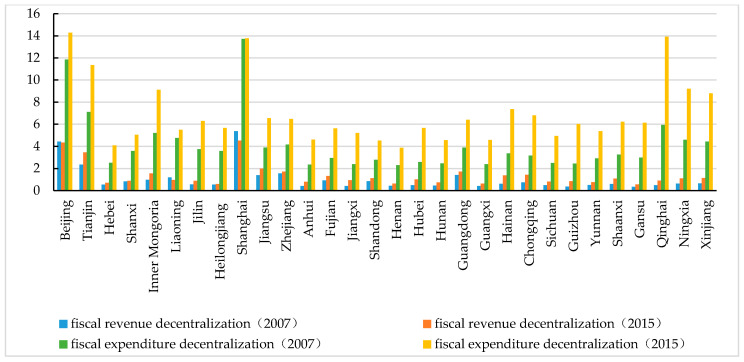
China’s fiscal decentralization in 2007 and 2015.

Figure 2 demonstrates that fiscal expenditure decentralization is far greater than fiscal revenue decentralization, which is in line with the characteristics of China’s fiscal reform since 1994. Regions such as Beijing, Tianjin and Shanghai are municipalities directly responsible to the central government of China, and thus enjoy higher degree of fiscal decentralization than other regions. However, provinces such as Inner Mongolia, Qinghai, Ningxia and Xinjiang also have high fiscal expenditure decentralization because the Chinese central government provides a large number of transfer payments to these minority areas [34]. The central government introduced the transfer payments in 2000 and these minority areas received CNY 70.4 billion transfer payments in 2017 [39]. There are also lots of restricted development zones in minority areas for ecological protection. Therefore, in order to maintain national stability and establish financial compensation mechanisms of ecological protection, minority areas receive more central government transfer payments than other areas. Except for Beijing and Shanghai, fiscal revenue decentralization of other provinces has experienced an increase. Fiscal expenditure decentralization has shown an upward trend for all provinces with the exception of Shanghai.

**Figure 3:**
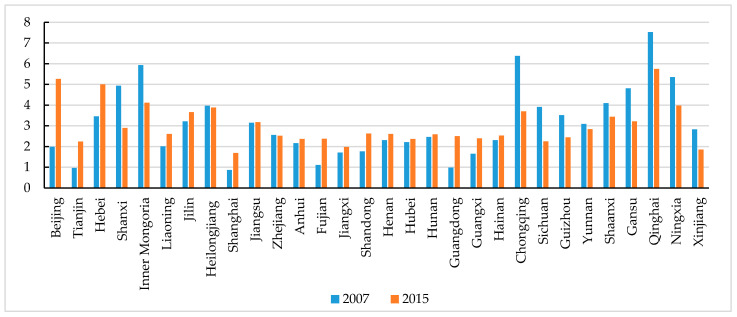
China’s government environmental preference in 2007 and 2015.

From 2007 to 2015, the proportion of environmental protection expenditure in local fiscal expenditure in China failed to exceed 8%, and it varies greatly across the provinces (Figure 3). The government environmental preference of Beijing showed a continuously upward trend, but the trend of the remaining regions is mixed.

In general, the empirical analysis is performed in four steps. Firstly, a fixed effect model is employed to test the direct effect of fiscal decentralization on the environment. Secondly, the product of the environmental preference and fiscal decentralization is an interaction term to test the moderating effect of government environmental preference in fixed effect models. Thirdly, a threshold model is used to test the variation the fiscal decentralization and environmental pollution, as the environmental preference jumps over the threshold levels. Lastly, an alternative explanatory variable, industrial nitrogen oxide (NO_X_) emission density, is used for the robustness check.

## 4. Empirical Test and Scientific Discussion

As described in Section 3, model (1) is estimated first to test hypothesis 1 (Section 4.1). Then model (2) is estimated to test hypothesis 2 (Section 4.2). Furthermore, model (3) is estimated to explore the threshold effect of government environmental preference (Section 4.3). These are followed by robustness check in Section 4.4 and scientific discussion in Section 4.5.

### 4.1. Results of Testing Hypothesis 1

We used fixed effect models to grasp the impact of fiscal decentralization on environmental pollution. Wald test results show that an individual-fixed effect model should be used rather than the pooled regression, and Hausman test results also support the individual-fixed effect (instead of the random effect) models. Columns in Table 2 show the results of both province-fixed effect and year-fixed effect models.

The estimation results show fiscal revenue decentralization plays a negative role in controlling environmental pollution, which confirms hypothesis 1. This conclusion is consistent with research results by others [22]. On the one hand, fiscal revenue decentralization represents the amount of taxes retained by local governments. In order to increase fiscal revenue, local governments may give more opportunities to high pollution and high energy consumption companies by relaxing environmental regulations. On the other hand, fiscal revenue decentralization stimulates economic growth [32]. Until 2015, China’s economy was still dominated by the secondary industry. Thus, economic development and environment protection have not yet achieved a win–win situation.

The coefficient of fiscal expenditure decentralization on the environment is significant in no-minority provinces. However, the magnitude of its effect is far smaller than that of fiscal revenue decentralization. This result is consistent with the results of He (2015) [21]. In China, although some government officials pursue GDP growth for the purpose of promotion, various policies, rules, and laws formulated by the central government affect the local governments in exercising their authority. From the strong time effect of industrial SO_2_ emission density, we can find that the central government has a strong control over the environment. The central government has designed 23 general public budget items for local governments and can directly intervene in the fiscal expenditure of local governments through various means, such as transfer payments. However, the information asymmetry between the central and local governments may lead to the inefficient use of transfer payments [32].

### 4.2. Results of Testing Hypothesis 2

We used fixed effect models to test the moderating effect of government environmental preference (Table 3). In general, the negative environmental effect of fiscal decentralization can be moderated by the government environmental preference (*GEP2*), which verifies Hypothesis 2. Although the efficiency of government environmental protection expenditure is low, increasing the share of environmental protection expenditure in total fiscal expenditure can improve environmental quality. World Bank research shows that the environment can be improved when the investment in pollution control accounts for 2.0% to 3.0% of a country’s GDP. Although the moderating effect of environmental preference is much smaller than the effect of fiscal revenue decentralization, the negative environmental effect of fiscal revenue decentralization can be offset, when the proportion of environmental protection expenditure reaches 10.21% (calculated from the regression coefficient). In terms of fiscal expenditure decentralization, the moderating effect of government environmental preference (GEP2) is significant in no-minority provinces.

Nevertheless, in terms of per capita fiscal environmental protection expenditure (GEP1), the estimated coefficient of government environmental preference interaction is not significant, which is consistent with empirical results by Zhang (2018) [9]. Therefore, the threshold effect will be examined next.

### 4.3. Further Analysis: Panel Threshold Modeling Results

After the analysis of the estimation results of the above fixed effect models, we now use per capita fiscal environmental protection expenditure as a threshold variable to explore the impact of the government environmental preference (*GEP1*) on the relationship between fiscal decentralization and environmental pollution. The threshold effect model also considers province-fixed effects and time-fixed effects. The threshold effect test and the threshold values are obtained after 300 bootstrap iterations in estimating each equation. The results are shown in Table 4. Regardless of the use of fiscal revenue or expenditure decentralization as the independent variable, the double threshold effect is significant at the 1% level, and the two threshold values are 1.102 and 3.510, which correspond to CNY 110.2 and 351 per capita fiscal environmental protection expenditure.

The estimation results for the threshold effect of government environmental preference (*GEP1*) represented by per capita fiscal environmental protection expenditure are shown in Table 5. Like the fixed effect model above, fiscal revenue decentralization has a significantly positive coefficient implying a negative environmental effect. However, as government environmental preference exceeds the threshold value, the negative environmental effect of fiscal revenue decentralization gradually weakens. Thus Hypothesis 2 is verified through the threshold effect models. This result is similar to the conclusion of Hong et al. (2018) [28]. It indicates that environmental protection expenditure works effectively, when the growth rate of government environmental protection expenditure is greater than that of total fiscal expenditure. Because of the existence of positive externalities of environmental protection, only when the fiscal environmental protection expenditure is high enough so that the private marginal income of environmental protection becomes greater than the social marginal income, relevant players will rationally choose to protect the environment, and the fiscal environmental protection expenditure is effective.

In terms of fiscal expenditure decentralization, the estimated coefficient in the whole samples is significant only when the per capita fiscal environmental protection expenditure is less than CNY 110.2. However, in no-minority provinces, government environmental preference can alleviate the negative effect of fiscal expenditure decentralization on reducing environmental pollution to a certain extent. Therefore, from the perspective of expenditure, the result based on non-minority provinces support hypothesis 2.

**Figure 4:**
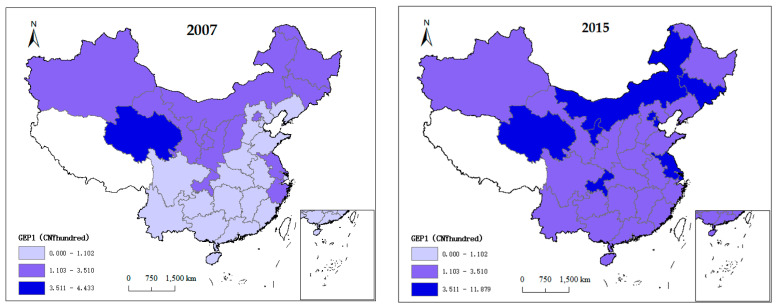
Classification of China’s provinces by thresholds in 2007 and 2015.

According to the above threshold model estimates, we clustered the 30 provinces based on their government environmental preference in 2007 and 2015. The results are shown in Figure 4. From 2007 to 2015, most of China’s provinces had crossed the higher threshold. This implies that different regions should manage government environmental protection expenditures differently [8]. Provinces that have not crossed the second threshold need to increase their per capita fiscal environmental protection expenditures to at least CNY 351. However, regions that have exceeded the second threshold need to take measures to mitigate the negative environmental effect of fiscal decentralization by focusing on environmental supervision and raising the proportion of environmental protection spending in local fiscal expenditure as mentioned above.

### 4.4. Robustness Check

For the robustness check, we used an alternative explanatory variable, industrial nitrogen oxide (NO_X_) emission density which is defined as the ratio of industrial NO_X_ emission over the land area. NO_X_ emission is one of the major industrial air pollutants and an important air pollutant monitoring indicator. Industrial NO_X_ emission data are from the China Environmental Statistics Yearbook.

The robustness estimation results for fixed effect models in Table 6 show that the environmental effect of fiscal revenue decentralization is negative, consistent with the estimation results of the fixed effect model in Section 4.1, but the magnitude of the impact of fiscal revenue decentralization on industrial NO_X_ emission density is smaller than that on industrial SO_2_ emission density. The direct effects of fiscal expenditure decentralization on the environment are still significant in non-minority provinces.

From a revenue perspective, the moderating effect of government environmental preference (represented by per capita fiscal environmental protection expenditure) still does not pass the *t*-test, while the moderating effect of government environmental preference (represented by the proportion of environmental protection in local fiscal expenditure) is significant (Table 7). The direction of impact is also consistent with the results in Section 4.2. In terms of fiscal expenditure decentralization, the moderating effect of government environmental preference (represented by the proportion of fiscal environmental protection expenditure) are still significant in non-minority provinces.

The robustness test results of the threshold effect model in Table 8 show that the single threshold effect is significant. The sign of the estimated coefficient is consistent with that from the model in Section 4.3.

The estimated results of the threshold models with industrial NO_X_ emission density as the explanatory variable are shown in Table 9. From the perspective of fiscal revenue, when the per capita fiscal environmental protection expenditure is less than CNY 351, the estimated coefficient of fiscal decentralization is 3.824 which is significant at the level of 1%. When it is greater than CNY 351, the impact of fiscal decentralization on environmental pollution becomes weaker and statistically insignificant. From the perspective of fiscal expenditure, the estimated coefficients of fiscal decentralization are not significant. However, the single threshold effect of fiscal expenditure decentralization in non-minority areas is significant, and when per capita environmental protection expenditure is greater than CNY 351, the magnitude of the negative environmental effect of fiscal expenditure decentralization is reduced significantly. This result is consistent with the findings in Section 4.3. 

### 4.5. Scientific Discussion

The empirical studies in federal countries [17,40] support the first generation of fiscal decentralization theory that decentralization allows policies to be more tailored to local conditions [11]. However, the results of this paper verify the second generation of fiscal decentralization theory which assumes that the local governments be essentially “rational people” [14], which is consistent with existing research in developing countries [19]. Electoral control and yardstick competition are two opposing forces that shape a non-monotonic effect of fiscal decentralization on public sector efficiency [41]. Different from the electoral system in some developed countries, Chinese officials are appointed by upper-level officials, which encourages local governments to be in favor of pursuing high economic growth motivated by the central government [42,43].

The results of this paper provide empirical evidence from China as a somewhat basis for study in environmental federalism in developing countries. This article suggests that developing countries should develop some certain sustainability-oriented policies when facing economic and environmental choices. Given that fiscal decentralization has a positive impact on economic growth [32], developing countries can alleviate the negative environmental effects of fiscal decentralization by increasing environmental preference [44]. Evidently, this would create a win–win situation for the economy and environment eventually.

In addition, due to the difficulty of obtaining data, we cannot conduct a cross-country comparative study like Sigman (2014) [40]. In the future, we will compare further the differences in the environmental effect of fiscal decentralization among developed and developing countries. Moreover, it should be noted that this paper selects the density of air pollutants as an indicator of environmental pollution based on the environmental carrying capacity, which may lead to results slightly biased, although the seven provinces in minority areas are further removed from the sample. A variety of indicators can be selected to measure environmental pollution more properly for more robust results.

## 5. Conclusions

In order to investigate the relationship between fiscal decentralization and environmental pollution, we use a fixed effect model and a threshold model separately to test the environmental effect of fiscal decentralization and the role of government environmental preference in China’s 30 provinces. This paper has three main conclusions. First, fiscal decentralization has a negative impact on reducing environmental pollution. With the exclusion of minority provinces, the environmental effect of fiscal decentralization becomes more significant. Second, the magnitude of the negative effect of fiscal expenditure decentralization is far smaller than that of fiscal revenue decentralization. Third, government environmental preferences can alleviate the negative environmental effect of fiscal decentralization to a certain extent. Specifically, the negative environmental effect of fiscal decentralization can be moderated by fiscal environmental protection expenditure per capita. In addition, as the proportion of fiscal environmental protection expenditure in total fiscal expenditure exceeds the threshold value, the negative environmental effect of fiscal revenue decentralization gradually weakens.

This research has important implications for sustainability-oriented policy making. First, the central government can reduce the degree of fiscal revenue decentralization in economically developed regions and encourage local governments to achieve a better balance between economic development and environmental protection. Controlling revenue decentralization and increasing expenditure decentralization enable fiscal decentralization to play a role in stimulating economic growth and causing less environmental pollution.

Second, it is important to increase local government environmental preference to reduce the negative impact of fiscal revenue decentralization on the environment. Local governments cannot blindly increase fiscal revenue by relaxing the environmental standards of enterprises and the level of government environmental supervision. Government environmental preferences can alleviate the negative environmental effect of fiscal decentralization, which can be used as one of the incentives to encourage governments to improve environmental preferences.

Finally, this study also sheds light on the amount of fiscal environmental protection expenditure per capita and its proportion in the total fiscal expenditure of local governments. On the one hand, local governments need to improve the fiscal expenditure structure. China should firmly and continuously expand the scale of fiscal environmental protection expenditure and increase its proportion in total fiscal expenditure. The growth rate of fiscal environmental protection expenditure should not be lower than that of total fiscal expenditure. Specifically, local governments ought to increase the proportion of environmental protection expenditures to about 10% in order to offset the adverse impact of fiscal decentralization on the environment. On the other hand, when local governments increase per capita fiscal environmental protection expenditure to more than CNY 350, the negative environmental impact of fiscal decentralization can be minimized. Consequently, the government could carry out differentiated environmental management for different regions. The regions with per capita fiscal environmental protection expenditures less than approximately CNY 350 ought to increase environmental protection expenditures to minimize the negative impact of fiscal revenue decentralization on the environment. Regions with per capita fiscal environmental protection expenditures exceeding CNY 350 need to improve environmental quality through some other environmental management means. More environmental protection indicators should be incorporated into the assessment standards and promotion mechanism of local officials, so that government environmental preference improves.

## Figures and Tables

**Table 1:** Description of the variables.

Variables	Description	Mean	SD	Min	Max
EP	Industrial SO_2_ emission/land area (tons/km^2^)	5.349	6.848	0.163	57.723
FD1	Per capita fiscal revenue of local government/per capita fiscal revenue of central government	1.189	0.983	0.348	5.379
FD2	Per capita fiscal expenditure of local government/per capita fiscal expenditure of central government	5.946	2.925	2.308	14.660
GEP1	Per capita fiscal environmental protection expenditure (CNY hundred)	2.368	1.625	0.315	11.879
GEP2	Fiscal environmental protection expenditure/total fiscal expenditure (%)	3.022	1.115	0.846	7.520
GDP	GDP per capita (CNY ten thousand)	3.212	1.900	0.654	10.320
FDI	Foreign direct investment/GDP (%)	2.409	1.871	0.068	8.198
ST	No of patents granted per 10,000 persons	5.976	8.030	0.350	43.312
IS	Value added of the secondary industry/total value-added (%)	47.746	7.906	19.738	61.500

**Table 2:** Estimation results for effects of fiscal decentralization.

Independent Variable	Full Sample		Excluding Minority Provinces	
	FD1	FD2	FD1	FD2
FD	7.234 ** (3.103)	0.751 (0.658)	9.670 *** (1.870)	2.693 ** (1.202)
GDP	−0.988 (3.898)	8.995 (7.252)	−4.927 (5.564)	4.298 (5.195)
GDP^2^	0.692 (0.514)	−0.288 (0.399)	1.182 ** (0.536)	0.181 (0.397)
FDI	−0.335 (0.300)	−0.063 (0.188)	−0.988 *** (0.300)	−0.844 ** (0.405)
ST	−0.202 * (0.107)	−0.158 * (0.087)	−0.133 (0.078)	−0.131 (0.082)
IS	0.086 (0.108)	0.093 (0.120)	0.027 (0.076)	0.037 (0.083)
_cons	−5.311 (8.216)	−16.513 (17.421)	0.256 (8.151)	−13.888 (12.329)
province fixed effects	Yes	Yes	Yes	Yes
time dummies	Yes	Yes	Yes	Yes
N	270	270	207	207

Robust standard errors, *p*-values are in parentheses under the coefficients. * *p* < 0.1, ** *p* < 0.05, *** *p* < 0.01.

**Table 3:** Estimation results for moderating effects of government environmental preference.

Independent Variable	Full Sample				Excluding Minority Provinces			
	FD1		FD2		FD1		FD2	
FD	6.177 ** (2.679)	6.218 ** (2.697)	1.334 (0.939)	0.932 (0.745)	7.975 *** (2.237)	8.168 *** (2.362)	1.334 * (1.197)	2.494 * (1.241)
GEP1_t-1_*FD	−0.277 (0.184)		−0.069 (0.048)		−0.205 (0.142)		−0.046 (0.036)	
GEP2_t-1_*FD		−0.609 * (0.345)		−0.082 (0.051)		−0.482 * (0.242)		−0.083 * (0.041)
GDP	−4.883 (5.048)	−3.197 (4.176)	−1.343 (4.333)	2.779 (3.244)	−9.361 (8.927)	−9.174 (8.871)	−5.538 (7.809)	−4.500 (7.914)
GDP^2^	1.173 * (0.590)	0.913 * (0.489)	0.485 (0.393)	−0.072 (0.360)	1.520 * (0.797)	1.434 * (0.769)	0.740 (0.542)	0.559 (0.538)
FDI	−0.228 (0.307)	−0.204 (0.284)	−0.091 (0.232)	−0.056 (0.197)	−0.756 ** (0.358)	−0.740 ** (0.339)	−0.681 * (0.391)	−0.677 * (0.384)
ST	−0.121 ** (0.051)	−0.122 ** (0.050)	−0.073 * (0.042)	−0.064 (0.045)	−0.057 (0.063)	−0.067 (0.059)	−0.042 (0.056)	−0.044 (0.055)
IS	0.085 (0.110)	0.080 (0.108)	0.085 (0.113)	0.070 (0.105)	0.085 (0.100)	0.074 (0.100)	0.072 (0.090)	0.070 (0.089)
_cons	−0.186 (5.640)	−0.770 (4.810)	−3.424 (6.867)	−5.683 (7.461)	7.165 (10.939)	7.725 (10.851)	1.457 (9.408)	1.105 (9.368)
province fixed effects	Yes	Yes	Yes	Yes	Yes	Yes	Yes	Yes
time dummies	Yes	Yes	Yes	Yes	Yes	Yes	Yes	Yes
N	240	240	240	240	184	184	184	184

Robust standard errors, *p*-values are in parentheses under the coefficients. * *p* < 0.1, ** *p* < 0.05, *** *p* < 0.01.

**Table 4:** Results of the threshold effect test.

Independent Variable	Threshold	F Stat	Prob	Crit10	Crit5	Crit1	Threshold Estimator	
**Full sample**								
FD1	Single	140.51	0.000	25.262	35.009	63.286	Th-1	1.102
	Double	135.34	0.000	17.455	26.905	49.597	Th-21 Th-22	1.102 3.510
	Triple	105.42	0.113	111.896	134.894	203.689	Th-3	3.535
FD2	Single	106.55	0.000	25.261	33.256	52.899	Th-1	1.102
	Double	79.49	0.000	23.058	28.581	40.528	Th-21 Th-22	1.102 3.510
	Triple	53.70	0.253	66.119	77.026	94.912	Th-3	3.535
**Excluding minority provinces**								
FD1	Single	87.66	0.000	16.295	20.406	35.816	Th-1	3.510
	Double	59.87	0.000	12.751	16.843	29.619	Th-21 Th-22	1.102 3.510
	Triple	108.53	0.583	206.299	224.478	284.917	Th-3	3.535
FD2	Single	87.70	0.000	20.116	25.375	38.862	Th-1	1.102
	Double	66.34	0.000	17.892	24.083	35.044	Th-21 Th-22	1.102 3.510
	Triple	54.28	0.653	116.280	127.437	148.921	Th-3	3.535

**Table 5:** Estimation results for the threshold models.

Independent Variable	Threshold Interval	Full Sample		Excluding Minority Provinces	
		FD1	FD2	FD1	FD2
FD	(GEP1 ≤ 1.102)	8.295 *** (1.541)	1.828 ** (0.782)	9.794 *** (1.548)	2.799 *** (0.982)
	(1.102 < GEP1 ≤ 3.510)	6.049 *** (1.559)	1.022 (0.602)	8.237 *** (1.578)	2.017 ** (0.830)
	(GEP1 > 3.510)	4.264 *** (0.998)	0.554 (0.471)	6.472 *** (1.225)	1.531 ** (0.669)
GDP		−7.679 *** (2.673)	0.030 (2.971)	−10.687 ** (5.186)	−2.852 (2.579)
GDP^2^		1.479 *** (0.431)	0.634 ** (0.274)	1.837 *** (0.619)	0.905 ** (0.394)
FDI		−0.248 (0.197)	−0.066 (0.156)	−0.551 *** (0.190)	−0.224 (0.209)
ST		−0.134 *** (0.038)	−0.117 *** (0.033)	−0.078 (0.047)	−0.149 *** (0.045)
IS		0.060 (0.055)	0.106 (0.079)	0.039 (0.055)	0.122 (0.080)
_cons		4.437 (3.380)	−7.820 (8.209)	7.547 (5.422)	−6.761 (6.446)
province fixed effects		Yes	Yes	Yes	Yes
time dummies		Yes	Yes	Yes	Yes
N		270	270	207	207

Robust standard errors, *p*-values are in parentheses under the coefficients. * *p* < 0.1, ** *p* < 0.05, *** *p* < 0.01.

**Table 6:** Estimation results for effects of fiscal decentralization (pollutant: nitrogen oxide (NO_X_)).

Independent Variable	Full Sample		Excluding Minority Provinces	
	FD1	FD2	FD1	FD2
FD	2.202 *** (0.570)	0.363 (0.402)	3.573 *** (0.961)	1.745 ** (0.659)
GDP	1.649 (4.015)	3.714 (3.927)	−2.036 (3.962)	−3.302 (5.732)
GDP^2^	0.288 (0.422)	0.059 (0.411)	0.642 (0.504)	0.626 (0.612)
FDI	−0.184 (0.212)	−0.101 (0.195)	−0.490 * (0.267)	−0.537 * (0.301)
ST	−0.136 * (0.072)	−0.118 * (0.066)	−0.092 (0.056)	−0.085 (0.058)
IS	0.046 (0.107)	0.048 (0.110)	0.019 (0.097)	0.014 (0.090)
_cons	−3.769 (5.801)	−6.156 (6.288)	1.987 (4.158)	2.094 (4.824)
province fixed effects	Yes	Yes	Yes	Yes
time dummies	Yes	Yes	Yes	Yes
N	270	270	207	207

Robust standard errors, *p*-values are in parentheses under the coefficients. * *p* < 0.1, ** *p* < 0.05, *** *p* < 0.01.

**Table 7:** Estimation results for moderating effects of government environmental preference (pollutant: NO_X_).

Independent Variable	Full Sample				Excluding Minority Provinces			
	FD1		FD2		FD1		FD2	
FD	2.577 * (1.297)	2.344 ** (0.570)	1.141 * (0.651)	0.678 (0.519)	3.765 ** (1.681)	3.764 ** (1.531)	2.203 *** (0.745)	2.080 ** (0.767)
GEP1_t-1_* FD	−0.334 (0.234)		−0.086 * (0.049)		−0.310 (0.238)		−0.090 (0.061)	
GEP2_t-1_* FD		−0.597 * (0.234)		−0.117 * (0.063)		−0.565 * (0.316)		−0.147 ** (0.077)
GDP	−4.779 (7.631)	−2.195 (7.631)	−6.448 (8.836)	−1.477 (6.411)	−11.540 (10.758)	−10.324 (9.997)	−15.908 (12.989)	−13.662 (12.352)
GDP^2^	1.231 (1.005)	0.791 (1.005)	1.170 (0.935)	0.519 (0.600)	1.734 (1.245)	1.438 (0.993)	1.902 (1.265)	1.508 (1.067)
FDI	−0.187 (0.225)	−0.168 (0.225)	−0.213 (0.260)	−0.170 (0.232)	−0.482 (0.315)	−0.482 (0.297)	−0.605 * (0.333)	−0.602 * (0.321)
ST	−0.100 * (0.058)	−0.104 (0.058)	−0.098 (0.062)	−0.085 (0.058)	−0.028 (0.071)	−0.046 (0.075)	−0.030 (0.065)	−0.035 (0.067)
IS	0.091 (0.140)	0.080 (0.140)	0.089 (0.138)	0.072 (0.135)	0.097 (0.144)	0.099 (0.151)	0.095 (0.128)	0.097 (0.135)
_cons	1.318 (7.234)	1.318 (5.958)	3.039 (7.430)	0.270 (5.709)	12.374 (12.062)	12.714 (11.839)	16.323 (15.043)	15.573 (14.590)
province fixed effects	Yes	Yes	Yes	Yes	Yes	Yes	Yes	Yes
time dummies	Yes	Yes	Yes	Yes	Yes	Yes	Yes	Yes
N	240	240	240	240	184	184	184	184

Robust standard errors, *p*-values are in parentheses under the coefficients. * *p* < 0.1, ** *p* < 0.05, *** *p* < 0.01.

**Table 8:** Results of the threshold effect test (pollutant: NO_X_).

Independent Variable	Threshold	F stat	Prob	Crit10	Crit5	Crit1	Threshold Estimator	
**Full sample**								
FD1	Single	158.10	0.000	25.297	39.239	94.247	Th-1	3.510
	Double	−31.52	1.000	20.743	41.584	118.515	Th-21 Th-22	3.227 3.637
FD2	Single	106.33	0.000	21.041	29.135	62.301	Th-1	3.510
	Double	−19.86	1.000	21.740	28.010	38.898	Th-21 Th-22	3.227 3.637
**Excluding minority provinces**								
FD1	Single	139.48	0.000	13.607	27.008	48.670	Th-1	3.510
	Double	176.61	0.000	13.628	18.795	33.543	Th-21 Th-22	3.535 3.535
FD2	Single	123.94	0.000	16.081	25.662	49.220	Th-1	3.510
	Double	50.32	0.003	16.077	19.670	26.322	Th-21 Th-22	3.535 3.570

**Table 9:** Estimation results for the threshold models (pollutant: NO_X_).

Independent Variable	Threshold Interval	Full Sample		Excluding Minority Provinces	
		FD1	FD2	FD1	FD2
FD	(GEP1 ≤ 3.510)	3.824 *** (1.104)	1.114 (0.657)	2.279 ** (0.885)	5.038 *** (1.001)
	(GEP1 > 3.510)	1.261 (1.031)	0.511 (0.422)	1.477 ** (0.708)	2.392 ** (1.120)
GDP		−6.433 (7.742)	−3.259 (7.849)	6.406 * (3.514)	−14.481 * (8.244)
GDP^2^		1.641 * (0.821)	1.128 (0.850)	−0.209 (0.257)	2.293 * (0.890)
FDI		−0.170 (0.202)	−0.107 (0.218)	−0.179 (0.208)	−0.303 (0.189)
ST		−0.128 *** (0.037)	−0.114 *** (0.043)	−0.156 *** (0.055)	−0.084 ** (0.034)
IS		0.074 (0.094)	0.087 (0.114)	0.056 (0.084)	0.078 (0.087)
_cons		1.788 (7.954)	−3.109 (7.467)	−14.434 (9.585)	13.413 (8.425)
province fixed effects		Yes	Yes	Yes	Yes
time dummies		Yes	Yes	Yes	Yes
N		270	270	207	207

Robust standard errors, *p*-values are in parentheses under the coefficients. * *p* < 0.1, ** *p* < 0.05, *** *p* < 0.01.
